# The role of OsMSH4 in male and female gamete development in rice meiosis

**DOI:** 10.1093/jxb/erv540

**Published:** 2015-12-28

**Authors:** Chaolong Wang, Yang Wang, Zhijun Cheng, Zhigang Zhao, Jun Chen, Peike Sheng, Yang Yu, Weiwei Ma, Erchao Duan, Fuqing Wu, Linglong Liu, Ruizhen Qin, Xin Zhang, Xiuping Guo, Jiulin Wang, Ling Jiang, Jianmin Wan

**Affiliations:** ^1^National Key Laboratory for Crop Genetics and Germplasm Enhancement, Jiangsu Plant Gene Engineering Research Center, Nanjing Agricultural University, Nanjing 210095, PR China; ^2^National Key Facility for Crop Gene Resources and Genetic Improvement, Institute of Crop Science, Chinese Academy of Agricultural Sciences, Beijing 100081, PR China

**Keywords:** Chiasmata, *Oryza sativa*, OsMSH4/OsMSH5 heterodimer, RPA complex, Trisomic.

## Abstract

Meiosis is essential for gametogenesis in sexual reproduction in rice (*Oryza sativa* L.). We identified a MutS-homolog (MSH) family gene *OsMSH4* in a trisomic plant. Cytological analysis showed that developments of both pollen and embryo sacs in an *Osmsh4* mutant were blocked due to defective chromosome pairing. Compared with the wild type, the *Osmsh4* mutant displayed a significant ~21.9% reduction in chiasma frequency, which followed a Poisson distribution, suggesting that class I crossover formation in the mutant was impaired. Temporal and spatial expression pattern analyses showed that *OsMSH4* was preferentially expressed in meiocytes during their meiosis, indicating a critical role in gametogenesis. Subcellular localization showed that OsMSH4–green fluorescent protein was predominantly located in the nucleus. OsMSH4 could interact with another MSH member (OsMSH5) through the N-terminus and C-terminus, respectively. Direct physical interaction between OsMSH5, OsRPA1a, OsRPA2b, OsRPA1c, and OsRPA2c was identified by yeast two-hybrid assays and further validated by pull-down assays. Our results supported the conclusion that the OsMSH4/5 heterodimer plays a key role in regulation of crossover formation during rice meiosis by interaction with the RPA complex.

## Highlight

The OsMSH4/OsMSH5 heterodimer interacts with type A and C RPA heterotrimer complexes during second-end capture to regulate crossing over during meiosis I in rice.

## Introduction

Meiosis is a key biological process in sexual reproduction which generates haploid gametes by a single round of DNA replication and is followed by two successive rounds of nuclear division. Meiosis I is a reductional division in which homologous chromosomes pair, synapse, recombine, and segregate; and meiosis II involves the separation of sister chromatids (Zickler and Kleckner, 1999; Ma, 2006). Homologous chromosome recombination (HR) is initiated by a double strand break (DSB) in one of two participating chromosomes, to form crossovers (COs). At this stage, a large number of DSBs occur, but only some of them are repaired to form COs, the others form non-crossovers (NCOs) (Youds and Boulton, 2011). There are two alternative means of CO formation giving rise to class I and class II COs in most eukaryotes (Zalevsky *et al.*, 1999; de Los *et al.*, 2003; Housworth and Stahl, 2003). As class I COs are sensitive to genetic interference, a relatively large distance is maintained between them, whereas class II COs are randomly distributed due to insensitivity to genetic interference (Mezard *et al.*, 2007; Osman *et al.*, 2011).

ZMMs (an acronym for yeast proteins Zip1/Zip2/Zip3/Zip4, Msh4/Msh5, Mer3) are a group of evolutionarily conserved proteins that play crucial roles in regulating CO formation, for example *Caenorhabditis elegans* (Kelly *et al.*, 2000), human (Tanaka *et al.*, 2006), mouse (Kneitz *et al.*, 2000), *Arabidopsis thaliana* (Higgins *et al.*, 2004), and rice (*Oryza sativa* L.) (Wang *et al.*, 2009; Luo *et al.*, 2013). In budding yeast, ZMMs are essential for the formation of class I COs (Borner *et al.*, 2004; Lynn *et al.*, 2007). Msh4 and Msh5, key factors in class I CO formation, form a functional heterodimeric complex to stabilize DNA structure in DNA repair and are important for the formation of double-Holliday junctions (Bocker *et al.*, 1999; Snowden *et al.*, 2004, 2008; Lynn *et al.*, 2007). Arabidopsis homologs of *MSH4* (*AtMSH4*) and *MSH5* (*AtMSH5*) exhibit extensive interdependent co-location to chromatin during prophase I, suggesting that these two proteins act together as a dimer to stabilize recombination intermediates and to promote formation of COs (Higgins *et al.*, 2004, 2008). In rice, OsMSH4 and OsMSH5 pair with each other to promote formation of class I COs, and neither *Osmsh4* nor *Osmsh5* mutants exhibit fertility (Luo *et al.*, 2013; Zhang *et al.*, 2014). However, the means by which the OsMSH4/OsMSH5 heterodimer regulates CO formation in plants are still unknown.

Replication protein A (RPA) is a single-stranded DNA-binding protein that is required for multiple processes in eukaryotic DNA metabolism, including DNA replication, DNA repair, and recombination (Wold, 1997; Iftode *et al.*, 1999). RPA is a heterotrimeric protein complex comprising three subunits, RPA1, RPA2, and RPA3; and, in most eukaryotic cells, each subunit has only a single RPA protein (Wold, 1997). However, plant genomes generally contain multiple copies of *RPA* genes; for example, *A. thaliana* has five putative *RPA1* genes, with two copies each of *RPA2* and *RPA3*. According to recent reports, both *AtRPA1C* and *AtRPA1A* (*AtRPA1A* perhaps replaces the role of *AtRPA1C* in its absence) play primary roles in initiation of HR events during meiosis (Osman *et al.*, 2009; Takashi *et al.*, 2009; Aklilu *et al.*, 2014).

Rice has three copies of *RPA1* and *RPA2*, and one of *RPA3* (Ishibashi *et al.*, 2006; Shultz *et al.*, 2007). These RPAs can be divided into three types of RPA complex: RPA1a–RPA2b–RPA3 (type A), RPA1b–RPA2a–RPA3 (type B), and RPA1c–RPA2c–RPA3 (type C) (Ishibashi *et al.*, 2006). These different complexes may have differentiation and redundancy functions. The *osrpa1a* mutant showed unformed embryo sacs and abnormal chromosome fragmentation in male meiocytes after anaphase I, indicating that *OsRPA1a* has an essential role in meiotic DSB repair. OsRPA2c, another subunit of RPA2, is essential for promoting wild-type levels of class I COs in partnership with OsRPA1c (Chang *et al.*, 2009; Li *et al.*, 2013). Until now, neither the mechanism of how the RPA complex controls CO formation nor the functional relationship between the RPA complex and ZMM protein has been fully characterized.

We identified an *Osmsh4* mutant among the progeny of a trisomic plant. The *OsMSH4* locus, isolated by map-based cloning, encodes a ZMM protein, which can interact with OsMSH5 to form a heterodimer that stabilizes the formation of class I COs during second-end capture. Our results indicate that *OsMSH4* is preferentially expressed during meiosis and is essential for promoting crossing over.

## Materials and methods

### Plant materials and growth conditions

**Fig. 1.**
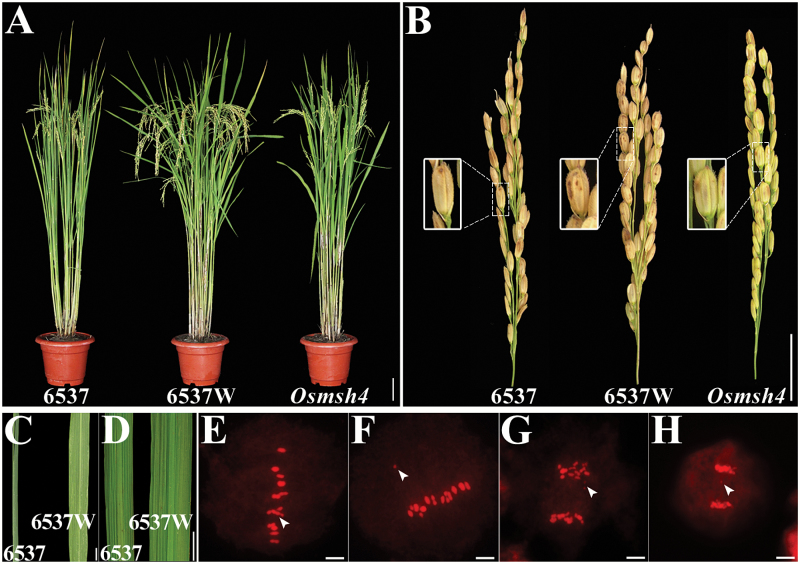
Comparison of trisomic (6537), wild-type (6537W), and *Osmsh4* mutant plants. (A) Mature plant morphologies of 6537, 6537W, and *Osmsh4* mutant plants. (B) Panicles of 6537, 6537W, and the *Osmsh4* mutant. (C, D) Leaves of 6537 and 6537W. (E–H) Meiotic metaphase I (E, F) and anaphase I (G, H) in 6537 showing one trivalent (E) and univalent chromosome (F–H) (arrowhead). Scale bar=10cm in (A), 5cm in (B), 1cm in (C) and (D), and 5 µm in (E–H).

A trisomic plant (named 6537) displaying abnormal seed setting was identified from anther culture of autotetraploid rice. Among the self-pollinated progeny, there was a high proportion of completely sterile plants (designated as *Osmsh4*), as well as a small number of normal plants (named as wild type) (Fig. 1). Among 419 progeny from plant 6537, we recorded segregation of 111 6537-like:308 *Osmsh4*-like individuals, close to 1:3. Self-pollinated progeny of a 6537-like plant segregated in a similar manner (Supplementary Fig. S1 at *JXB* online). An F_2_ population was generated from a cross between 6537 and 93-11 (ssp. *indica*) in order to fine-map the gene. All rice materials were grown in a paddy field in Hainan province (110°E, 18°N) or at the Chinese Academy of Agricultural Sciences, Beijing (116°E, 40°N) in the rice-growing season. All materials were planted with a spacing of 16.5×19.8cm, and a wide-row spacing of 23.5cm was set between the plots.

### Preparation of embryo sacs

About 1000 wild-type and mutant florets were fixed in FAA solution [18:1:1 (v/v) mixture of formalin, 70% ethanol, and acetic acid], and dissected ovaries were hydrated sequentially in 50, 30, and 15% ethanol and distilled water, stained with 1% eosin-Y for 8h, and washed in distilled water until colorless. Samples were pre-treated for 8h in citric acid–disodium hydrogen phosphate buffer (0.1mol l^–1^, pH 5.0) followed by Hoechest staining (25 ºC in darkness for 24h). They were rinsed three times with distilled water, and processed through an ethanol series (30, 50, 70, 90, and 100%) for dehydration; they were then treated in 1:1 ethanol and methylsalicylate for 1h, and cleared three times in methylsalicylate. The ovaries were finally examined using a laser confocal scanning microscope (Zeiss Microsystems LSM 700).

### Meiotic chromosome examination

Young panicles (40–60mm) of both the wild type and the mutant were fixed in Carnoy’s solution (ethanol:glacial acetic 3:1). First, one of the six anthers was stained with 1% aceto carmine (Sigma-Aldrich Chemical) to gauge the developmental stage for optical microscopy. Anthers at the appropriate stages were squashed under a cover slip in 40% acetic acid. After freezing in liquid nitrogen for 5min, cover slips were removed and samples were dried at room temperature before treatment with 20 µl of 0.1mg ml^–1^ propidium iodide for 20min to stain chromatin. The male meiocytes were observed using a fluorescence microscope (Leica DM5000B). Images were captured using a Leica Application Suite 3.3, merged, and enhanced using Photoshop CS (Adobe).

### Scanning (SEM) and transmission electron microscopy (TEM)

Anthers from the wild type and the *Osmsh4* mutant were fixed in 2.5% glutaraldehyde for 24h, rinsed three times using distilled water, dehydrated through an ethanol series, fixed in 1% OsO_4_ for 2h, again dehydrated through an ethanol series, and subjected to critical point drying with CO_2_. The anthers were coated with gold by E-100 ion sputter and observed with a scanning electron microscope (S3400; Hitachi). For TEM, mature anthers were fixed in 1% glutaraldehyde and 1% OsO_4_ for 1h and dehydrated through an ethanol series. The samples were embedded in Spurr’s medium prior to thin sectioning. Sections were double-stained with 2% uranyl acetate and 2.6% aqueous lead citrate solution, and examined with a JEM-1230 transmission electron microscope (Jeol) at 80kV.

### RNA *in situ* hybridization

Young spikelets were fixed overnight in a FAA (RNase-free) fixative solution at 4 ºC, followed by dehydration in an alcohol series of ethanol and xylene, and then embedded in paraffin (Paraplast Plus, Sigma). An *OsMSH4* cDNA fragment was amplified with primer O4-Insitu (Supplementary Table S3) and cloned into the pGEM-T Easy vector (Promega). The probe was then transcribed *in vitro* using a DIG Northern Starker Kit (Cat. no. 2039672, Roche) following the manufacturer’s instructions. RNA hybridization and immunological detection were performed according to a protocol described by Kouchi and Hata (1993).

### β-Glucuronidase (GUS) histochemical staining

A putative 2.8kb genomic promoter fragment upstream of the ATG start codon was amplified by PCR, and the fragment was cloned into the binary vector pCAMBIA1305 to drive *GUS* reporter gene expression. GUS staining of young T_1_ generation spikelets from transgenic plants was performed according to a previously described method (Jefferson, 1987).

### Quantitative real-time reverse transcription–PCR (qRT–PCR)

Total RNA was extracted using an RNeasy Plant Mini Kit (Qiagen). First-strand cDNA was synthesized with a QuantiTect Reverse Transcription Kit (Qiagen) using 1 µg of RNA. For qRT–PCR, 0.4 µl of cDNA, 0.2 µM of gene-specific primers, and SYBR Premix Ex Taq Kit (TaKaRa) were mixed into 20 µl reaction volumes for PCR performed with an ABI Prism 7900 HT Sequence Detection System (Applied Biosystems) following the manufacturer’s instructions. The rice *ubiquitin* gene was used as an internal control. PCRs in two independent biological replicates were carried out in triplicate for each sample.

### Yeast two-hybrid (Y2H) assay

The coding regions of genes were amplified and cloned into the Y2H prey vector pGADT7 or bait vector pGBDT7 (Clontech). The pGADT7 and pGBDT7 constructs were then co-transformed into Gold yeast (*Saccharomyces cerevisiae*) following the Yeast Handbook (Clontech). The resultant yeast strains were grown on SD–Leu/–Trp plates for 3 d at 30 ºC. For each pair of interaction tests, five individual clones were mixed in 60 µl of 0.9% NaCl and diluted 10-, 100-, and 1000-fold. The dilution series were then spotted on the selective media -LTH (SD–Leu/–Trp/–His) or -LTHA (SD–Leu/–Trp/–His/–Ade) containing X-α-gal (40 µg ml^–1^).

### Pull-down assay

For *in vitro* pull-down assays, coding regions of *OsMSH5*, native *OsMSH4*, mutated *Osmsh4*, *OsRPA1a*, *OsRPA2b*, *OsRPA1c*, and *OsRPA2c* were cloned into pGEX4T-1 or pMAL-c2X vectors to generate *OsMSH5*-*GST*, *OsRPA2c*-*GST*, native *OsMSH4-MBP*, mutated *Osmsh4-MBP*, *OsRPA1a-MBP*, Os*RPA2b-MBP*, *OsRPA1c*-*MBP*, and *OsRPA2c*-*MBP* plasmids. The proteins including fusions and empty tags were expressed in *Escherichia coli* BL21 cells (TransGen). Glutathione *S*-transferase (GST)-, OsRPA2C–GST-, and OsMSH5–GST-coupled beads were used to capture OsRPA1a–maltose-binding protein (MBP), OsRPA2b–MBP, OsRPA1c–MBP, and OsRPA2c–MBP. MBP-, Osmsh4–MBP-, and OsMSH4–MBP-coupled beads were used to capture OsMSH5–GST. The pull-down analyses were performed as described (Miernyk and Thelen, 2008) and detected with horseradish peroxidase-conjugated anti-MBP and anti-GST monoclonal antibodies (1:2000; New England Biolabs).

## Results

### Cytological analysis of trisomic line 6537

Compared with the wild type (a disomic plant, named 6537W), trisomic 6537 plants featured low seed setting (average 24.1%), with narrower and darker green leaves as well as slightly more slender grains (Fig. 1A–D). As trisomic 6537 originated among the progeny of anther-cultured autotetraploid rice, we surveyed meiotic cells to determine the chromosome constitution of 6537. At metaphase I, in most cases (>90% of metaphase I cells), there were 11 bivalents and a trivalent, or 12 bivalents (as in the wild type) and an unpaired univalent outside the equatorial plate (Fig. 1E, F). At anaphase I, the extra chromosome either went to one of the two daughter cells of the dyad, or, in most cases, was lost as a laggard chromosome (Fig. 1G, H). We deduced that 6537 was a typical trisomic line.

### Characterization of male and female gametes in the *Osmsh4* mutant

**Fig. 2.**
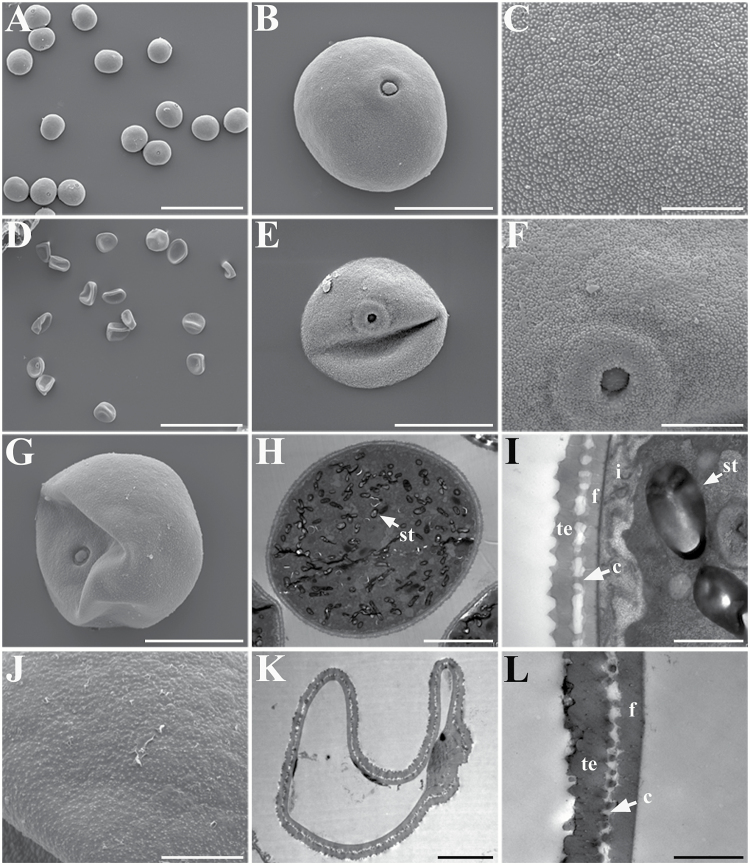
Scanning electron micrography and transmission electron micrography of pollen from the wild type and *Osmsh4* mutant. (A) Wild-type pollen grains. (B) Magnified single pollen grain from (A). (C) Futher magnification showing the exine from (B). (D) *Osmsh4* microspore. (E and G) Two types of pollen grains in the *Osmsh4* mutant. (F and J) Magnified pollen exines from (E) and (G). (H) Transmission electron micrograph image showing wild-type pollen. (I) Magnification of the pollen wall from (I). (K) Transmission electron micrograph showing the *Osmsh4* pollen. (L) Magnification of the pollen wall from (K). st, starch; i, intine; f, foot layer; c, columella; te, tectum. Scale bars=100 µm in (A) and (D), 20 µm in (B), (E), and (G), 5 µm in (C), (F), and (J), 10 µm in (H), 5 µm in (K), and 1 µm in (I) and (L).

Mutant *Osmsh4* plants isolated from the selfed progeny of trisomic line 6537 had no obvious differences from the wild type except for sterility (see the Materials and methods) (Supplementary Table S1). In order to explain the sterility of the *Osmsh4* mutant, we investigated its pollen and embryo sac development. Histochemical staining showed that pollen grains in the mutant were rarely stained by 1% I_2_–KI solution, in contrast to 100% staining in the wild type, suggesting abortion of pollen in the mutant (Supplementary Fig. S2A, B). We then compared the anthers of the mutant and the wild type by SEM and TEM. Anthers of the *Osmsh4* mutant were shorter and smaller than those of the wild type (Supplementary Fig. S3A, B), but with no structural differences in the epidermis (Supplementary Fig. S3C–H). Mature pollen grains from the wild type were spherical (Fig. 2A, B) and filled with starch grains (Fig. 2H), and the pollen exine was covered with sporopollenin (Fig. 2C). Pollen grains of the *Osmsh4* mutant, however, were irregular, either smaller (Fig. 2E) or larger than those of the wild type (Fig. 2G), coated with less sporopollenin (Fig. 2F, J), and contained no internal contents (Fig. 2K). In the wild type, the pollen wall was composed of exine and intine, and the exine was further divided into the tectum and foot layer which were linked by the columella (Fig. 2I), but the *Osmsh4* mutant formed abnormal intine and exine, with a thicker tectum and foot layer, and degraded columella (Fig. 2L).

**Fig. 3.**
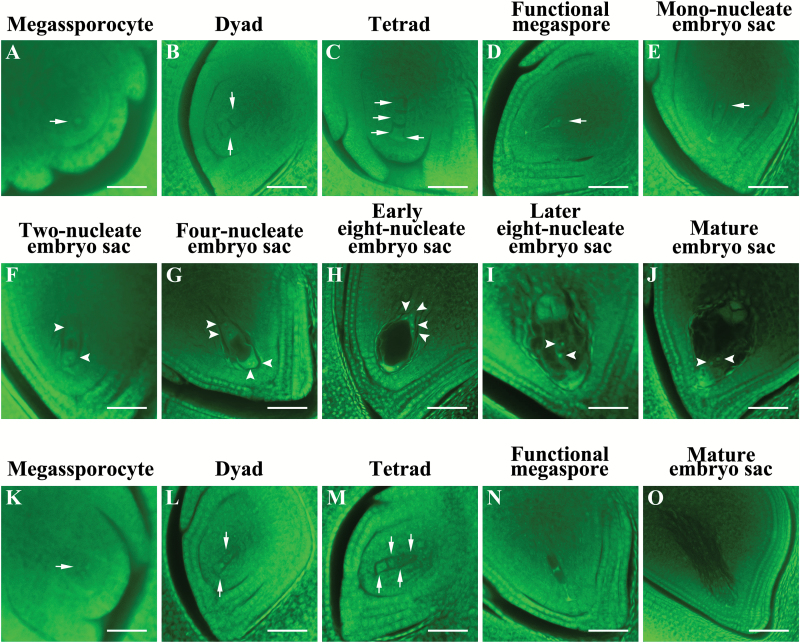
Embryo sac development in the wild type (A–J) and *Osmsh4* mutant (K–O). Nuclei during megasporogenesis (arrow) and megagametogenesis (arrowhead). Scale bar=20 µm.

The *Osmsh4* mutant failed to set seed when pollinated with wild-type pollen, suggesting complete female sterility. Generally, the normal mature embryo sac contains a group of antipodal cells, two synergid cells, an egg cell, and one central cell with two polar nuclei (Supplementary Fig. S2C, D). However, the *Osmsh4* mutant had empty embryo sacs with no cell differentiation (Supplementary Fig. S2E). Whole-mount stain-clearing laser scanning confocal microscopy (WCLSM) was then applied to compare the embryo sac development of the wild type and mutant at various developmental stages. In the wild type, megasporocytes underwent normal meiotic divisions to produce four megaspores (Fig. 3A–C), among which three located at the micropylar end degenerated, followed by enlargement of the remaining one that becomes the functional megaspore (Fig. 3D). Finally, the mononucleate embryo sac went through three rounds of mitosis to form an eight-nucleate embryo sac (Fig. 3E–J). Compared with the wild type, megasporocytes and dyads developed normally in the *Osmsh4* mutant (Fig. 3K, L). However, the megaspore near the chalaza was degenerated at the tetrad stage (Fig. 3M), resulting in failure to form functional megaspores (Fig. 3N) or mature embryo sacs (Fig. 3O). These results confirmed that both male and female gametes of the *Osmsh4* mutant were non-functional.

### Disrupted chromosome behavior in the *Osmsh4* mutant

To investigate male sterility in the sterile mutants further, we compared meiotic chromosomal behavior in pollen mother cells of both the wild type and *Osmsh4* mutant at different stages.

**Fig. 4.**
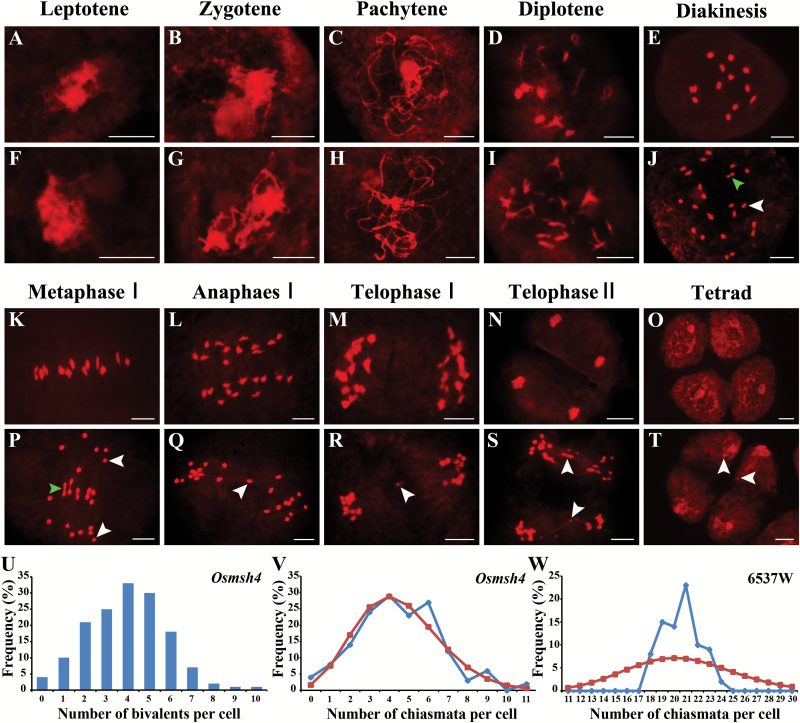
Meiotic chromosome dynamics of pollen mother cells in the wild type 6537W (A–E and K–O) and the *Osmsh4* mutant (F–J and P–T). (U) Frequencies of the number of bivalents per cell in the *Osmsh4* mutant. (V) Observed (blue rhombus) and predicted Poisson (red squares) distributions of chiasmata per cell in the *Osmsh4* mutant. (W) Observed (blue rhombus) and predicted Poisson (red squares) distributions of chiasmata per cell in the wild type 6537W. The green arrow in (P) points to the bivalent and the white arrow to the univalent. Scale bars=5 µm.

Condensing chromosomes in the wild type became clearly visible at leptotene (Fig. 4A); homologous chromosomes were partially synapsed and concentrated to one side of the nucleus at zygotene (Fig. 4B). During pachytene, full chromosome synapsis was evident with completion of the synaptonemal complex (SC) (Fig. 4C). At diplotene, full synapsis disassembled and homologous chromosomes remained paired at the chiasmata (Fig. 4D). The chromosomes then condensed further to form 12 bivalents at diakinesis (Fig. 4E), before aligning on the equatorial plate at metaphase I (Fig. 4K). Homologous chromosomes separated and migrated to separate poles during anaphase I and telophase I (Fig. 4L, M), thus reducing the chromosome number by a half. During the second division, sister chromatids separated and a tetrad was formed, each cell with 12 chromosomes (Fig. 4N, O).

Compared with the wild type, chromosome behavior in the *Osmsh4* mutant showed no differences at the leptotene, zygotene, and pachytene stages (Fig. 4F–H). However, many homologous chromosomes separated from each other at diplotene (Fig. 4I, see below). During diakinesis, abnormal pairing became more apparent, with the presence of many univalents as well as normal bivalents (Fig. 4J), a typical desynaptic phenotype. During metaphase I, the unpaired univalents dispersed throughout the nucleus, while the remaining bivalents aligned on the equatorial plane (Fig. 4P). From anaphase I to metaphase II, two types of chromosome aberration were observed, namely delayed chromosome segregation (Fig. 4Q, R) and random univalent segregation (Supplementary Fig. S4A–C), both of which can lead to unequal chromosome numbers in the two daughter cells. We also noticed bridge formation at telophase II (Fig. 4S) and micronuclei at the tetrad stage (Fig. 4T). Sister chromatids also separated asynchronously during anaphase II and telophase II (Supplementary Fig. S4D, E), resulting in triad formation (Supplementary Fig. S4F). Thus multiple aberrations in microspore development led to complete pollen sterility in the *Osmsh4* mutant.

### Random distribution of residual chiasmata in the *Osmsh4* mutant

It is widely acknowledged that chiasmata play a critical role in the stability of bivalents (Ma, 2006). In order to investigate differences in CO between the *Osmsh4* mutant and the wild type, we quantified the chiasmata frequencies at diakinesis. The *Osmsh4* mutant had a dramatically reduced number of bivalents when compared with the wild type (Fig. 4J). Statistical analysis indicated a mean bivalent frequency in the *Osmsh4* mutant as low as 3.95 per cell, in sharp contrast to 12 per cell in the wild type. Fewer than 10 bivalents per cell were common in the *Osmsh4* mutant; indeed, nine and 10 bivalents were observed only in single cells of the mutant (Fig. 4U). According to criteria previously described by Sanchez *et al.* (2001), rod-shaped bivalents were scored as having one chiasma whereas ring bivalents had two. Mean chiasmata number in the *Osmsh4* mutant ranged from 0 to 11, averaging 4.51 per cell (*n*=152), compared with 20.58 (*n*=81) for the wild type. Thus, the bivalent number in the *Osmsh4* mutant was greatly reduced relative to the wild type. The number of remaining chiasmata per cell in the *Osmsh4* mutant was consistent with a Poisson distribution (Fig. 4V), indicating that chiasmata were distributed randomly among cells, whereas the distribution in the wild type deviated significantly from a Poisson model (Fig. 4W). These results suggested that formation of class I COs was disrupted in the *Osmsh4* mutant.

### Isolation of the *OsMSH4* gene

Nineteen normally fertile plants were selected from 1360 self-pollinated progeny of 6537 plants (Supplementary Fig. S1). Among them, 14 gave progeny that segregated for fertility and sterility in 3:1 ratios [e.g. one set of data was 218 fertile:65 sterile (χ^2^_3;1_=0.62, *P*_2df_>0.05], indicating that sterility was caused by a single recessive nuclear gene. The remaining five plants displayed normal seed setting (>80% fertile) and were regarded as wild type.

To identify the *OsMSH4* gene, we used a map-based cloning approach to construct a segregating population with trisomic 6537 as female and 93-11 as male parents. Among 47 F_1_ plants, 12 with narrow, dark-green leaves and slender grains were discarded as probable trisomics. The remaining 35 individuals with normal phenotype were self-pollinated and the F_2_ progeny were used as a mapping population.

**Fig. 5.**
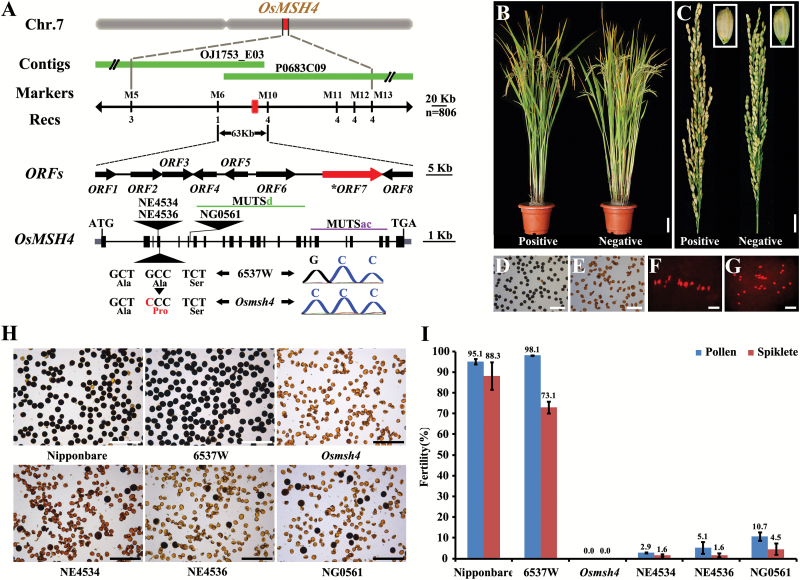
Identification and complementation of *OsMSH4*. (A) The *OsMSH4* gene was narrowed to a 63kb region between InDel markers M6 and M10. OJ1753_E03 and P0683C09 are BAC and PAC clones covering the locus. Eight ORFs were predicted in the critical region, and ORF7 was identified as *OsMSH4*. The mutant sequence of *Osmsh4* has a single nucleotide change, guanine (G) to cytosine (C), in the fourth exon. The three *TOS17* insertions are indicated by triangles. (B) Growth morphologies of positive and negative T_1_ plants. (C) Comparison of seed setting in positive and negative T_1_ plants. (D–G) Pollen and meiotic metaphase I of complemented lines (D and F) and negative transgenic T_1_ plants (E and G). (H) Pollen of Nipponbare, the wild type, the *Osmsh4* mutant, and three *TOS17* insertion lines. (I) Fertility scores of Nipponbare, the wild type, the *Osmsh4* mutant, and three *TOS17* insertion lines. Scale bars=10cm in (B), 2cm in (C), 200 µm in (D), (E), and (H), and 5 µm in (F) and (G).

We initially mapped the *OsMSH4* locus to a genomic region on chromosome 7L between Indel markers M6 and M10. Based on the reference sequence of cv. Nipponbare (http://rgp.dna.affrc.go.jp/E/IRGSP/index.html), we found a 63kb genomic region containing the *OsMSH4* locus spanned by BAC (bacterial artificial chromosome) clone OJ1753_E03 and PAC (plasmid P1-derived artificial chromosome) clone P0683C09. According to the Rice Genome Annotation Database (http://rapdb.dna.affrc.go.jp/), eight putative open reading frames (ORFs) were present in the region (Supplementary Table S2). To deduce which ORF was a possible candidate gene for *OsMSH4*, we sequenced the entire 63kb regions of the wild type and the mutant, and identified a single nucleotide substitution in gene *LOC_Os07g30240*. Using rapid amplification of cDNA ends (RACE) PCR, four transcript types of *LOC_Os07g30240* were detected (Supplementary Fig. S5), and the longest coding cDNA contained 24 exons, encoding a predicted protein consisting of 798 amino acids. The substitution at codon 415(G/C) in the fourth exon of *LOC_Os07g30240* caused an amino acid change from Ala139, which is highly conserved in plants, to proline (Fig. 5A; Supplementary Fig. S6). Sequence analysis showed that *LOC_Os07g30240* was predicted to encode a peptide with MUTSd (residues 195–536) and MUTSac domains (residues 551–738) (http://smart.embl-heidelberg.de/). To verify further that *LOC_Os07g30240* was the *OsMSH4* gene, transgenic plants were generated by introducing a 14.5kb wild-type *LOC_Os07g30240* genomic fragment into homozygous *Osmsh4* mutant plants. Positive transgenic T_1_ plants displayed normal growth morphologies and seed settings (Fig. 5B, C; Supplementary Fig. S7A, B). In addition, pollen development and chromosome pairing were significantly improved compared with mutant control plants (Fig. 5D–G). We also identified three lines of *TOS17* insertions in the *LOC_Os07g30240* gene and they, like the mutant, showed almost complete male and female sterility (Fig. 5H, I; Supplementary Fig. S7C). These results demonstrated that *LOC_Os07g30240* was the *OsMSH4* gene.

### Expression of *OsMSH4* during meiosis and subcellular localization

**Fig. 6.**
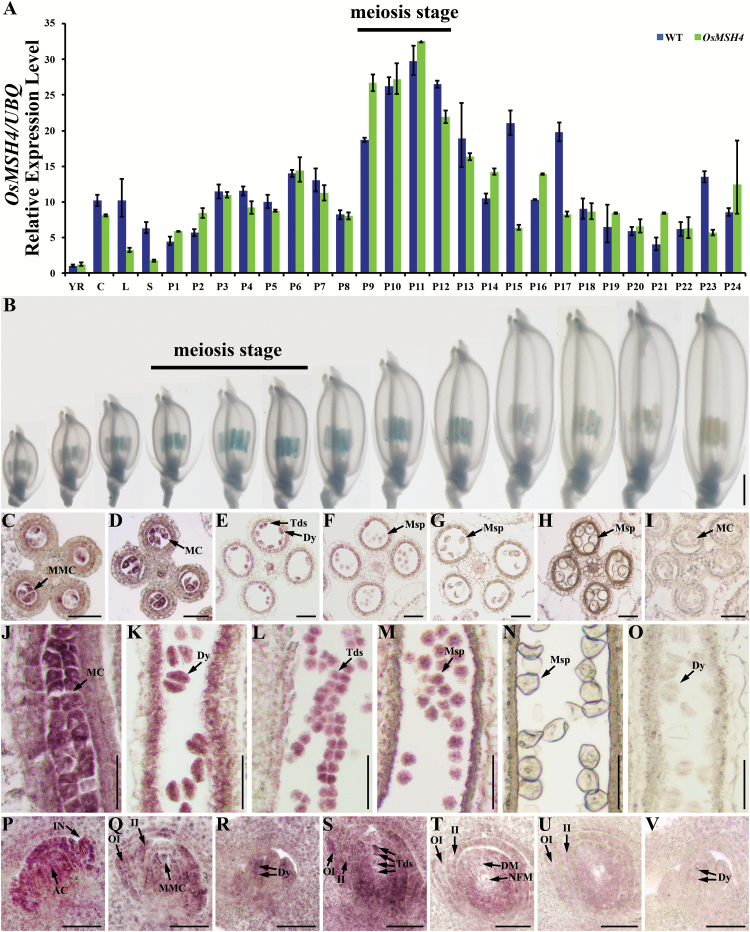
Expression pattern analysis of *OsMSH4*. (A) Temporal and spatial expression patterns of *OsMSH4* determined by quantitative RT–PCR. (B) GUS staining of panicles at various developmental stages. (C–H) *In situ* analysis of *OsMSH4* expression by transverse sections of an anther at the archesporial cell stage (C), microspore mother cell stage (D), dyad and tetrad stages (E), early microspore stage (F), middle microspore stage (G), and late microspore stage (H). (J–N) *In situ* analysis of *OsMSH4* expression by longitudinal anther sections at the microspore mother cell stage (J), dyad stage (K), tetrad stage (L), early microspore stage (M), and late microspore stage (N). (P–U) *In situ* analysis of *OsMSH4* expression in longitudinal sections of the embryo sac at the early megasporocyte stage (P), late megasporocyte stage (Q), meiotic stages (R, S), mononucleate embryo sac stage (T), and embryo sac mitosis stage (U). (I), (O), and (V) Negative controls with the sense probe of the anther at the microspore mother cell (I), dyad (O), and embryo sac at meiotic (V) stages. YR, young root; C, mature culm; L, mature leaf; S, mature sheath; P1–P24, spikelet lengths from 1cm to 24cm; MMC, megaspore mother cell; MC, meiotic cell; Dy, dyad cell; Tds, tetrads; Msp, microspore; AC, archesporial cell; IN, integument primordium; OI, outer integument; II, inner integument; DM, degenerated megaspore; NFM, non-functional megaspore. Scale bars=1mm in (B) and 50 µm in (C–V).

To examine the tissue specificity and developmental expression patterns of *OsMSH4*, we analyzed *OsMSH4* transcript levels by qRT–PCR. *OsMSH4* was weakly expressed in vegetative tissues, including young roots, mature culms, leaves, and leaf sheaths. However, developing spikelets showed dynamic and strong *OsMSH4* expression, which peaked at the meiosis stage (Fig. 6A). In general, the transcript pattern and levels of *OsMSH4* in the mutant showed no change relative to the wild type (Fig. 6A), indicating that the completely sterile phenotype in the *Osmsh4* mutant was not caused by transcriptional differences. We subsequently generated transgenic rice plants carrying an *OsMSH4*_pro_:*GUS* vector and found that GUS signals preferentially accumulated in the anthers of young panicles and peaked at the meiosis stage before declining and finally disappearing (Fig. 6B). To elucidate further the spatial and temporal expression patterns of *OsMSH4*, we performed RNA *in situ* hybridization with wild-type floral sections. The expression of *OsMSH4* was first observed at the archesporial cell stage (Fig. 6C) and was enhanced at the microscope mother cell stage (Fig. 6D, J; Supplementary Fig. S8A). Expression was slightly reduced at the dyad, tetrad, and early microspore stages (Fig. 6E, F, K–M; Supplementary Fig. S8B, C). Finally, only negligible *OsMSH4* signals could be detected at the middle and late microspore stages (Fig. 6G, H, N; Supplementary Fig. S8D). These results indicated that *OsMSH4* was specifically expressed during the meiotic stages and preferentially expressed in meiocytes. In analyses of expression during embryo sac development, *OsMSH4* was detected in whole ovules, including the outer integuments, inner integuments, and embryo sacs (Fig. 6P–T), and, as expected, expression was very high during meiosis (Fig. 6P–S). Post-meiosis, the *OMSH4* signals gradually declined with the formation of functional megaspores (Fig. 6T) and had almost disappeared at the second division stage (Fig. 6U). These results indicate that *OsMSH4* plays a vital role in normal meiosis.

**Fig. 7.**
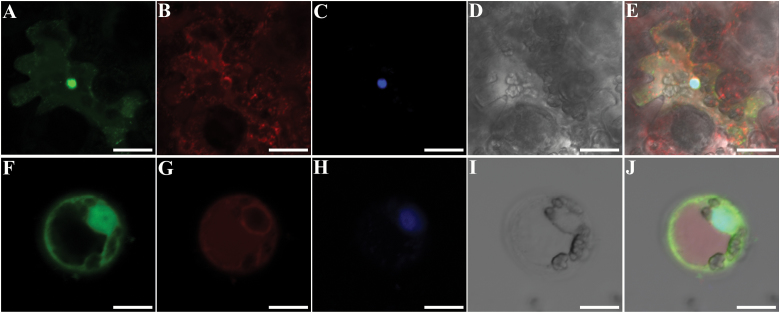
Subcellular localization of OsMSH4 protein in *N. benthamiana*. (A–E) Localization of the OsMSH4–GFP protein in leaf epidermal cells. (F–J) Localization of the OsMSH4–GFP protein in protoplasts. (A) and (F) OsMSH4–GFP is predominantly detected in the nucleus. (B) and (G) Localization of the ER marker. (C and H) DAPI staining. (D and I) Bright field images. (E and J) Merged images of (A–D) and (F–I), respectively. Scale bars=10 µm.

To determine the location of OsMSH4 protein, we constructed a fusion protein of the *OsMSH4* coding region and cDNA for green fluorescent protein (GFP). The *OsMSH4-GFP* fusion vector was driven by an octopine type Ti-plasmid right T-DNA gene 2′ promoter (TR 2′). As predicted, the OsMSH4–GFP fusion protein was predominantly localized in the nucleus (Fig. 7).

### The OsMSH4/5 heterodimer interacts with the type A and C RPA complex *in vitro*

**Fig. 8.**
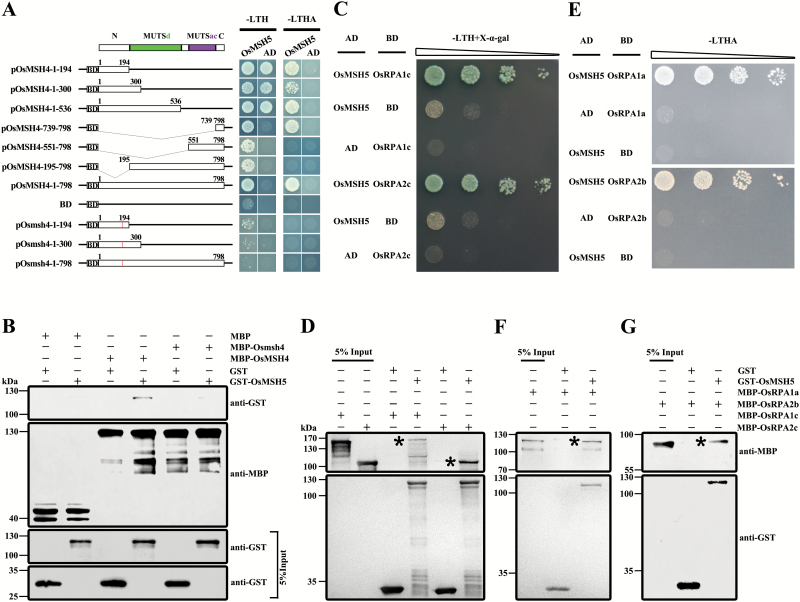
OsMSH5 interacts with OsMSH4, OsRPA1a, OsRPA2b, OsRPA1c, and OsRPA2c. (A) Yeast-two-hybrid (Y2H) assays to test the interaction region between OsMSH4 and OsMSH5. Constructs expressing different regions of *OsMSH4* and mutated *Osmsh4* were prepared in the bait vector pGBKT7 (BD) (left). Numbers indicate amino acid residues, and the red line indicates mutated amino acids. Full-length *OsMSH5* was cloned into the prey vector pGADT7 (AD). -LTH, selective medium (SD–Leu/–Trp/–His); -LTHA, selective medium (SD–Leu/–Trp/–His/–Ade). (B) *In vitro* pull-down assay of recombinant glutathione *S*-transferase (GST)–OsMSH5 using bead-coupled maltose-binding protein (MBP)–OsMSH4 and MBP–Osmsh4. (C) Y2H assay to test interactions between OsMSH5, OsRPA1c, and OsRPA2c. OsMSH5 was cloned into the prey vector pGADT7 (AD), and OsRPA1c and OsRPA2c were inserted in the bait vector pGBKT7 (BD). (D) *In vitro* pull-down assay of recombinant MBP–OsRPA1c and MBP–OsRPA2c using bead-coupled GST–OsMSH5. Asterisks indicate the full-length MBP–OsRPA1c and OsMBP–OsRPA2c proteins, respectively. (E) Y2H assay to test interactions between OsMSH5, OsRPA1a, and OsRPA2b. (F) *In vitro* pull-down assay of recombinant MBP–OsRPA1a using bead-coupled GST–OsMSH5. Asterisks indicate the full-length MBP–OsRPA1a. (G) *In vitro* pull-down assay of recombinant MBP–OsRPA2b using bead-coupled GST–OsMSH5. Asterisks indicate the full-length MBP–OsRPA2b.

A previous study showed that mouse Msh4 protein specifically interacts with Msh5 protein (Her *et al.*, 2001). Similarly, in humans, hMSH4 and hMSH5 interact at two distinct regions (Snowden *et al.*, 2004). However, no similar research has been reported in plants. We therefore investigated the interacting regions of OsMSH4 and OsMSH5. First, we cloned full-length *OsMSH5* into the prey vector pGADT7 (AD). Different regions of the wild-type and mutated *Osmsh4* gene in the MUTS domain were prepared as bait vectors pGBKT7 (BD). Y2H deletion assays indicated that both the N-terminus (1–194) and the C-terminus (739–798) of OsMSH4 could interact with OsMSH5. In addition, the interactions between truncated OsMSH4_551–798_ or OsMSH4_195–798_ proteins and OsMSH5 were reduced, probably resulting from peptide misfolding (Snowden *et al.*, 2004). However, the truncated proteins of mutated Osmsh4 (1–194 and 1–300) showed almost no detectable interaction with OsMSH5. Interestingly, the mutated Osmsh4 also could not interact with OsMSH5, even in the presence of the interactive C-terminus (739–798) (Fig. 8A). An *in vitro* pull-down assay confirmed that MBP–OsMSH4, but not MBP–Osmsh4, pulled down the GST–OsMSH5 fusion protein (Fig. 8B), thus confirming that OsMSH4 and OsMSH5 function as a heterodimer during class I CO formation.

Co-expression networks are highly efficient tools to predict protein–protein interactions (Lee *et al.*, 2004). By analyzing co-expression networks in rice, several genes involved in meiosis were reported to be co-regulated with *OsMSH4* and *OsMSH5* (Aya *et al.*, 2011). qRT–PCR showed that *OsMSH5* and *RPA2c* were highly co-expressed during the reproductive phase, especially at meiosis, and consistent with the prediction from the Rice Network. Furthermore, we found that *OsRPA1c* was a constitutively expressed gene (Supplementary Fig. S9). A Y2H assay that we conducted showed that OsMSH5, rather than OsMSH4, physically interacted with two subunits OsRPA1c and OsRPA2c of RPA (Fig. 8C). To prove the interactions further, we performed an *in vitro* pull-down assay. As in the Y2H assay, MBP–OsRPA1c and MBP–OsRPA2c showed specific affinity for GST–OsMSH5 (Fig. 8D). However, OsRPA1c also interacted with OsRPA2c (Supplementary Fig. S10). We also tested the interactions between OsMSH5 and other RPA family proteins by Y2H assays. As shown in Fig. 8E, strong interactions were detected between OsMSH5, OsRPA1a, and OsRPA2b, which were then confirmed by pull-down assays *in vitro* (Fig. 8F, G). These results suggested that OsMSH4, OsMSH5, OsRPA1a, OsRPA2b, OsRPA1c OsRPA2c, and OsRPA3 might act as a complex in meiosis I.

## Discussion

Eukaryotic genomes contain multiple MutS homologs (MSHs). With the exception of mitochondrial MSH1, eukaryotic MutS proteins are heterodimeric. For instance, MSH2 pairs with MSH6 or MSH3 to form MutSa and MutSb (Jiricny, 1998; Buermeyer *et al.*, 1999). hMSH4 and hMSH5 interact in both the N- and C-terminus (Snowden *et al.*, 2008). In general, MutS and its homologs can be divided into two lineages. The majority of prokaryotic and eukaryotic MutS proteins belong to the lineage that participates in DNA mismatch repair (MMR), prevents homologous DNA recombination in heterologous sequences, and mediates cell death induced by DNA-damaging agents (Matic *et al.*, 1996). The other lineage, including the MSH4/MSH5 dimer, is not functional in MMR, but promotes chromosome pairing and crossing over during meiosis (Nakagawa *et al.*, 1999). Homologs of MSH4 and/or MSH5 have been reported in different species (Ross-Macdonald and Roeder, 1994; Hollingsworth *et al.*, 1995; de Vries *et al.*, 1999; Edelmann *et al.*, 1999; Kelly *et al.*, 2000; Higgins *et al.*, 2004; Lu *et al.*, 2008; Luo *et al.*, 2013), and they have similar functions in promoting crossing over during meiosis in different species. In mice, disruption in the MutS homolog MSH5 showed a meiotic aberration, leading to both male and female sterility (de Vries *et al.*, 1999). In Arabidopsis, an *Atmsh4* mutant exhibited partial fertility and male meiotic defects (Higgins *et al.*, 2004), whereas the *Atmsh5* mutant showed developmental defects due to reduced chiasmata number in meiosis, thereby producing abnormal pollen and embryo sacs (Lu *et al.*, 2008). In rice, an *Osmsh5* mutant also exhibited abnormal meiosis and complete sterility (Luo *et al.*, 2013). A previous study showed that the interaction of OsMSH4 and OsMSH5, which played an earlier role than other ZMM proteins, was essential for CO formation in rice. Further, the loading of OsMSH5 depends on OsMSH4 as in Arabidopsis. Moreover, direct physical interaction detected between OsMSH5 and HEI10 (homolog of yeast Zip3) suggested an indirect interaction between ZMM proteins (Zhang *et al.*, 2014). However, neither the detailed interaction pattern of OsMSH4/OsMSH5 nor progression of CO formation triggered by OsMSH4/OsMSH5 heterodimer formation was explained. In our study, a missense mutation in a rice *Osmsh4* mutant caused substitution of Ala139 (A) by a proline (P) residue in *OsMSH4* (Fig. 5). Ala139 is located in a highly conserved region of MutS homologs throughout the plant kingdom (Supplementary Fig. S6). We speculate that this mutant, despite the existence of a normal C-terminus in the mutated Osmsh4, causes loss of interaction ability between Osmsh4 and OsMSH5, thereby resulting in complete male and female sterility in the *Osmsh4* mutant.

RPA plays essential roles in almost all DNA metabolic pathways. Multiple copies of RPA subunits occur in Arabidopsis and rice, but only one copy of each is present in yeast and most animals, indicating that RPA subunits have evolved diverse functions to regulate different DNA metabolic processes. In Arabidopsis, the *Atrpa1a* mutant defective in CO formation has a 60% reduction in chiasma frequency, but DNA DSBs are repaired, thus indicating that AtRPA1a has an important role in second-end capture during class I CO formation (Osman *et al.*, 2009). Another comprehensive and precise study of the RPA1 family suggested that the ACE (composed of RPA1A, RPA1C, and RPA1E) group is involved in DNA repair/recombination, whereas the BD (RPA1B and RPA1D) group promotes genomic DNA replication activities. In addition, RPA1C has a primary role in repair of DSB, a function that can be fulfilled by RPA1A in its absence (Aklilu *et al.*, 2014). It seems that the functions of RPA subunits in rice do not correspond to those of Arabidopsis. Namely, OsRPA1a might be the counterpart of AtRPA1C, given that all are possibly required in DSB repair, whereas the counterpart of AtRPA1A is OsRPA1c. However, analysis of double mutants to determine whether the RPA proteins in rice exhibit partial functional redundancy is still needed.

OsRPA1c and OsRPA2c promote meiotic crossing over; in *rpa2c* and *RPA1c* ^*RNAi*^, the chiasma frequency was reduced by ~78% and ~79%, respectively, and the distribution of remaining chiasmata conformed to Poisson distributions (Li *et al.*, 2013). Taking into account the *in vitro* studies using the RPA complex from yeast (Sugiyama *et al.*, 2006) and the high conservation of RPA among species, it seems reasonable to speculate that OsRPAs are also required for second-end capture during meiosis in rice. Our results indicated that OsMSH5 could interact with OsRPA1a, OsRPA2b, OsRPA1c, and OsRPA2c *in vitro*, suggesting that OsMSH4, OsMSH5, OsRPA1a, OsRPA2b, OsRPA1c, OsRPA2c, and OsRPA3 probably function as a complex in meiosis I. Based on the research of Snowden *et al.* (2004) and Sugiyama *et al.* (2006), we hypothesize that the OsMSH4/OsMSH5 heterodimer interacts with the type A and C RPA heterotrimeric complex to regulate or stabilize second-end capture during CO formation (Supplementary Fig. 11). These results suggest that ZMM proteins might be associated with other meiosis-related proteins, such as type A and C RPA complexes described in this study to co-ordinate CO formation.

The retention of a few chiasmata in the *Osmsh4* mutant suggested that a subset of chiasmata is OsMSH4 independent. This conclusion was supported by the finding that the residual chiasmata were distributed randomly among cells and among chromosomes, implying an absence of crossover interference. In *S. cerevisiae* there are two distinct classes of COs (Zalevsky *et al.*, 1999; Novak *et al.*, 2001), but only one exhibits interference. It was proposed that each kind of crossing over is promoted by a biochemically distinct pathway (de Los *et al.*, 2003). Class I events exhibiting interference were promoted by an MSH4/5-based complex, whereas class II events did not exhibit interference and were promoted by an MMS4/MUS81-based complex. In the *Osmsh4* mutant used in this study, the number of chiasmata was reduced to 21.9% of that of the wild type. However, the frequency of residual chiasmata that fit a Poisson distribution suggests that OsMSH4 is also required for class I CO formation.

Since Blakeslee *et al.* (1922) published the classical work on *Datura* trisomics, trisomy had been widely reported in plants, such as in tomato (Lesley, 1932; Rick and Barton, 1954; Hagemann, 1969) and rice (Iwata and Omura, 1984; Khush *et al.*, 1984; Wu *et al.*, 1988). In this study, we used a mutant derived from a trisomic plant to fine-map the sterility gene *Osmsh4*. On the one hand, the trisomic plants normally produced ~70% *Osmsh4* mutant progeny, much higher than the disomic heterozygous (only 25%) (Fig. 1; Supplementary Fig. S1). On the other hand, the trisomic plants were highly beneficial for preservation and functional studies of the sterile mutants. Plant breeders always attempt to combine favored alleles from different parents into elite varieties. Meiotic recombination helps to break the linked alleles to form new combinations; therefore, increasing the recombination frequency is generally beneficial for plant breeding (Wijnker and de Jong, 2008). Some genes involved in the recombination process in some plant species have been successfully isolated and manipulated for breeding improvement. For example, overexpression of *MutL homolog 1* (*MHL1*) in tomato led to a 10% increase in chiasma frequency (Puchta *et al.*, 1994), and overexpression of *RAD51* in Arabidopsis resulted in a 2-fold increase (Betzner *et al.*, 2004). A *Fanconi complementation group M* (*fancm*) mutant in *A. thaliana* had a 3-fold increase in CO frequency compared with the wild type (Crismani *et al.*, 2012). Inspired by these findings, it might be possible to mutate a *fancm* homolog in rice to increase the recombination frequency. Further study of the rice *OsMSH4* and *FANCM* genes may contribute to basic understanding of recombination and may be useful for enhancing genetic recombination in rice breeding.

## Supplementary data

Supporting data are available at *JXB* online.

Figure S1. Schematic strategy for developing genetic material from 6537.

Figure S2. Characterization of mature pollen and embryo sacs of the wild type (6537W) and *Osmsh4* mutant.

Figure S3. Scanning electron micrographs of anthers from the wild type and *Osmsh4*.

Figure S4. Meiotic chromosome dynamics of pollen mother cells in the *Osmsh4* mutant from anaphase I to the tetrad stage.

Figure S5. Schematic representation of four *OsMSH4* cDNA types.

Figure S6. Multiple sequence alignment of the amino acid sequence of OsMSH4 and its homologs.

Figure S7. Analysis of transgenic complementation and three lines of *TOS17* insertions.

Figure S8. *In situ* hybridization assays of *OsMSH4* at different stages of anther development.

Figure S9. Temporal and spatial expression pattern analyses of *OsMSH5*, *OsRPA1c*, and *OsRPA2c* by quantitative RT–PCR.

Figure S10. The interaction between OsRPA1c and OsRPA2c.

Figure S11. A schematic model depicting the OsMSH4/OsMSH5 heterodimer interacting with the OsRPA heterotrimeric complex during second-end capture to regulate crossover formation during meiosis I.

Table S1. Morphological trait comparison of the *Osmsh4* mutant with 6537 and 6537W.

Table S2. Gene annotations of eight ORFs within the 63kb region based on BAC clones of cv. Nipponbare.

Table S3. Primers used in this study.

Supplementary Data
