# Supplementary material for: The role of OsMSH4 in male and female gamete development in rice meiosis
Source: J Exp Bot. 2015 Dec 28;67(5):1447–59. doi: 10.1093/jxb/erv540 (PMC4762385; doi:10.1093/jxb/erv540)
Supplement: Supplementary Data [file supp_67_5_1447__index.html]

The role of OsMSH4 in male and female gamete development in rice meiosis — The role of OsMSH4 in male and female gamete development in rice meiosis — Supplementary Data 

# The role of OsMSH4 in male and female gamete development in rice meiosis

## Supplementary Data

Data files

- Supplementary\_figures\_S1\_S11\_tables\_S1\_S3.pdf - Supplementary Data
