# Supplementary material for: The role of OsMSH4 in male and female gamete development in rice meiosis
Source: J Exp Bot. 2015 Dec 28;67(5):1447–59. doi: 10.1093/jxb/erv540 (PMC4762385; doi:10.1093/jxb/erv540)
Supplement: Supplementary Data [file supp_erv540_Supplementary_figures_S1_S11_tables_S1_S3.pdf]

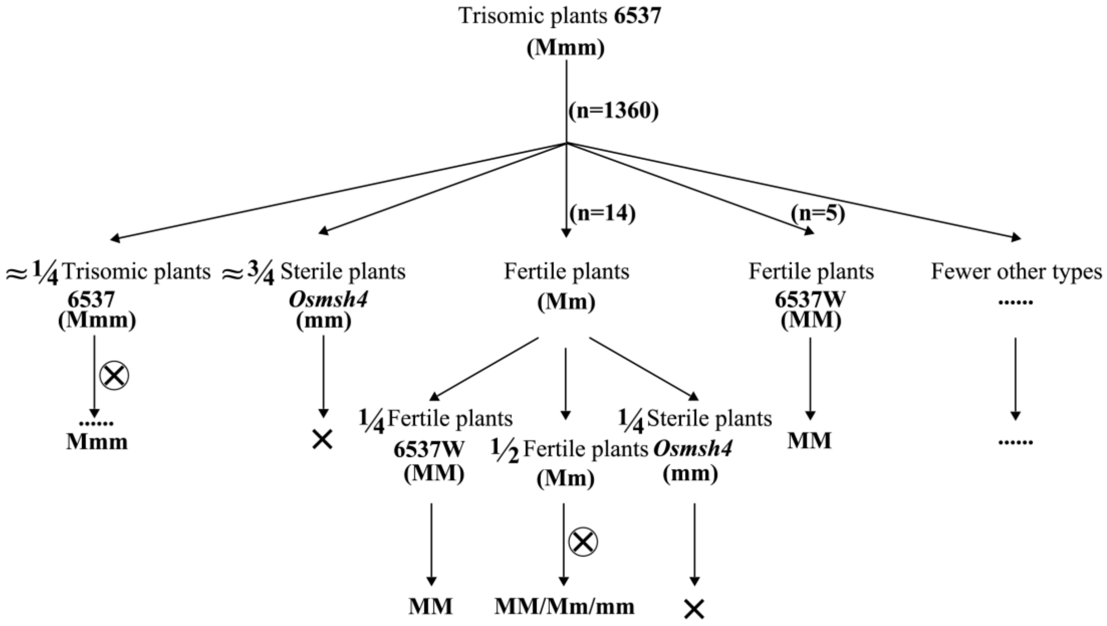

Supplementary Figure S1 Schematic strategy for developing genetic material from 6537. "×" indicates no progeny from homozygous recessive plants. "⊗" indicates self-pollination and repeat selection for maintaining the trisomic 6357 and heterozygous plants.

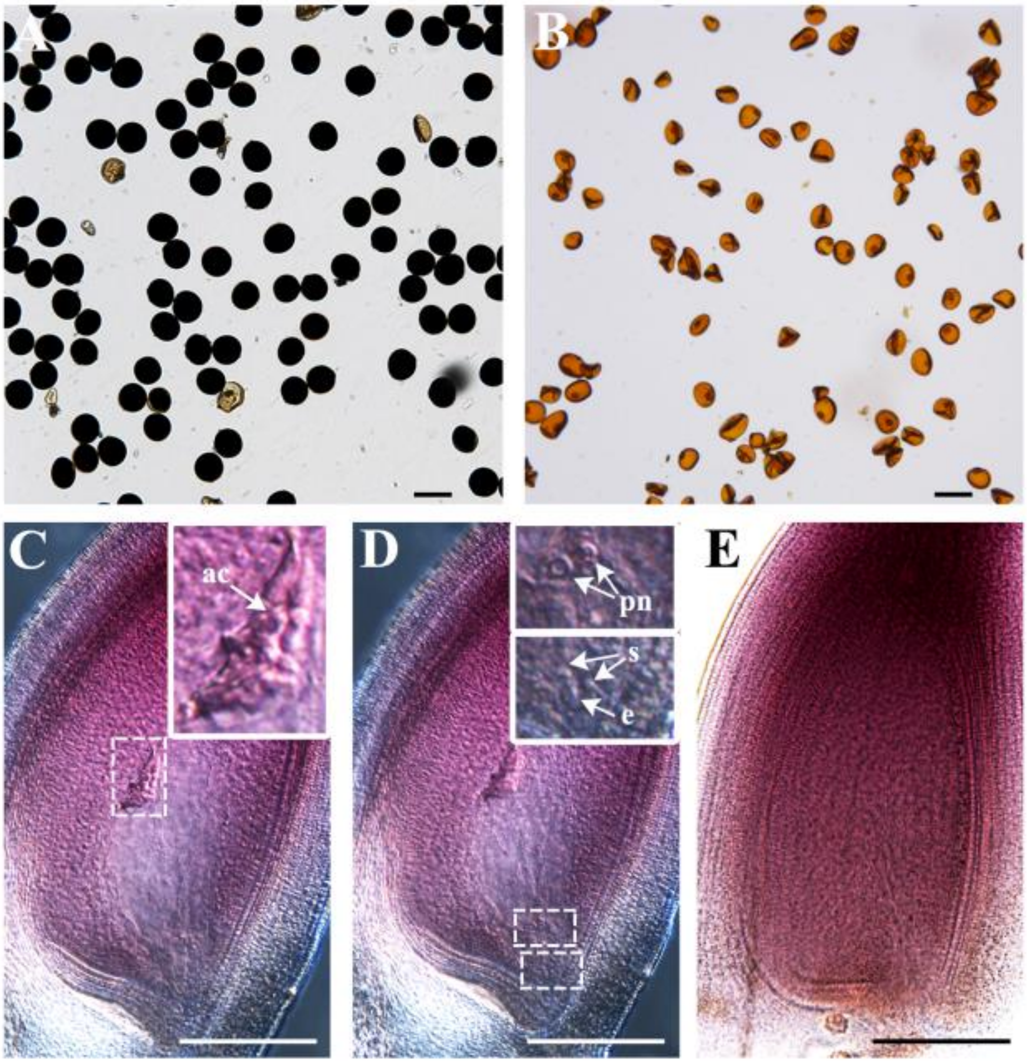

Supplementary Figure S2 Characterization of mature pollen and embryo sacs of the wild type (6537W) and *Osmsh4* mutant.

I_2-KI stained pollen of (A) wild type 6537W, and (B) *Osmsh4* mutant. (C) Normal embryo sac of the wild type 6537W contains a group of antipodal cells (ac). (D) Another focal plane of the embryo sac of 6537W showing one central cell with two polar nuclei (pn), two synergid cells (s) and a single egg cell (e). (E) An empty embryo sac from an *Osmsh4* mutant plant. Bars = 50 µm.

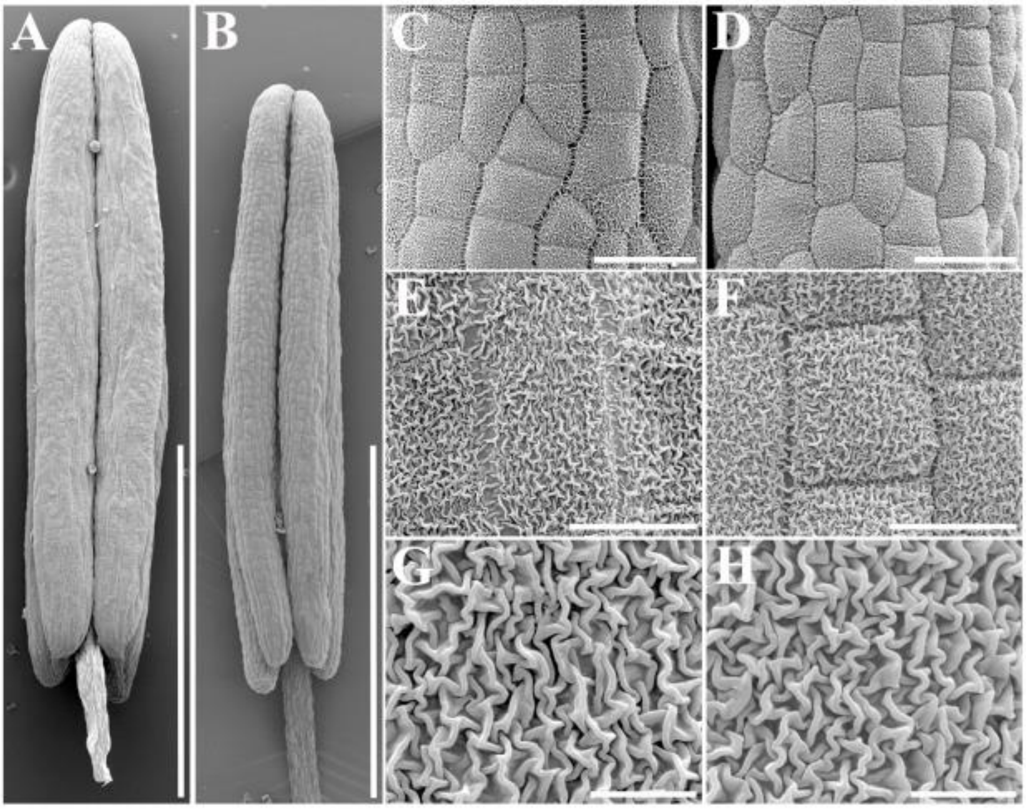

Supplementary Figure S3 Scanning electron micrographs of anthers from wild type and *Osmsh4*.

(A) Wild-type anther. (B) *Osmsh4* anther. (C), (E), (G) Higher magnification images from (A). (D), (F), (H) Higher magnification images from (B). Bars = 1 mm in (A) and (B), 50 µm in (C) and (D), 20 µm in (E) and (F), 5 µm in (G) and (H).

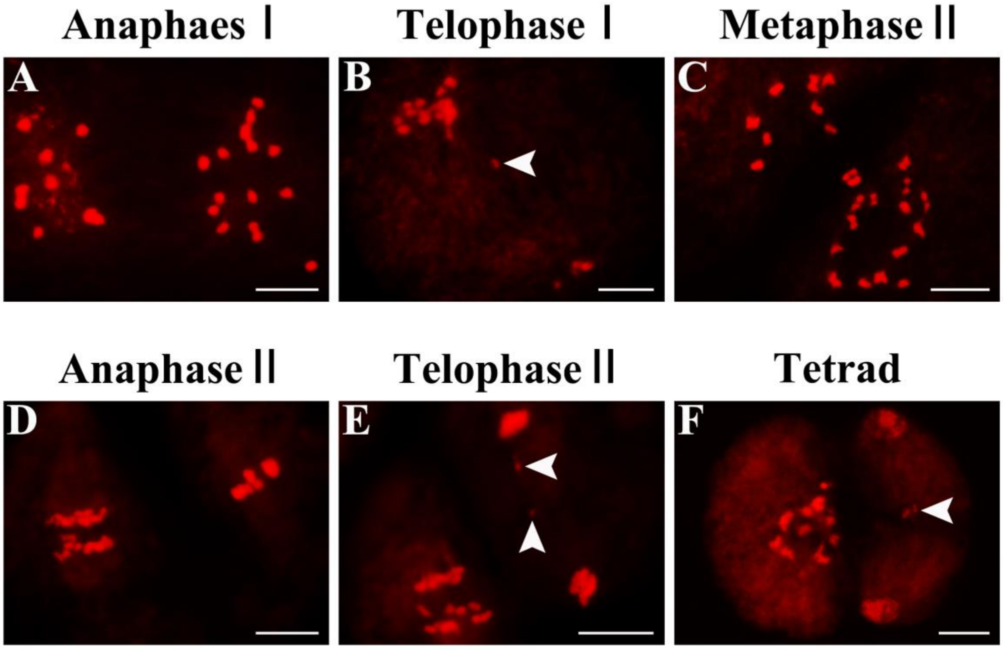

Supplementary Figure S4 Meiotic chromosome dynamics of pollen mother cells in *Osmsh4* mutant from anaphase I to the tetrad stage. White arrow points to the univalent. Bars = 5 µm.

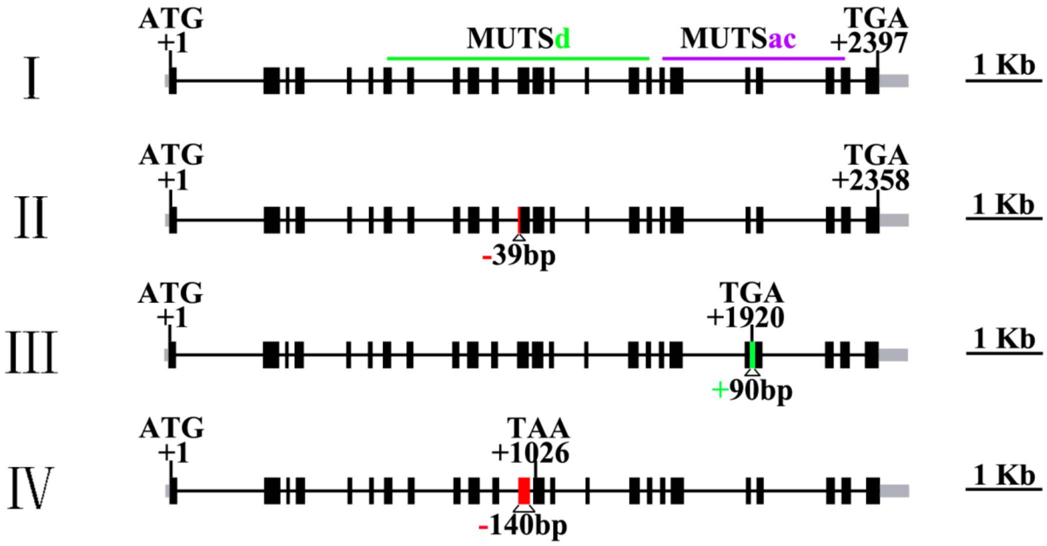

Supplementary Figure S5 Schematic representation of four *OsMSH4* cDNA types. Black boxes and lines indicate exons and introns; untranslated regions are shown in gray boxes. Green and red boxes indicate extra and less exons, respectively.

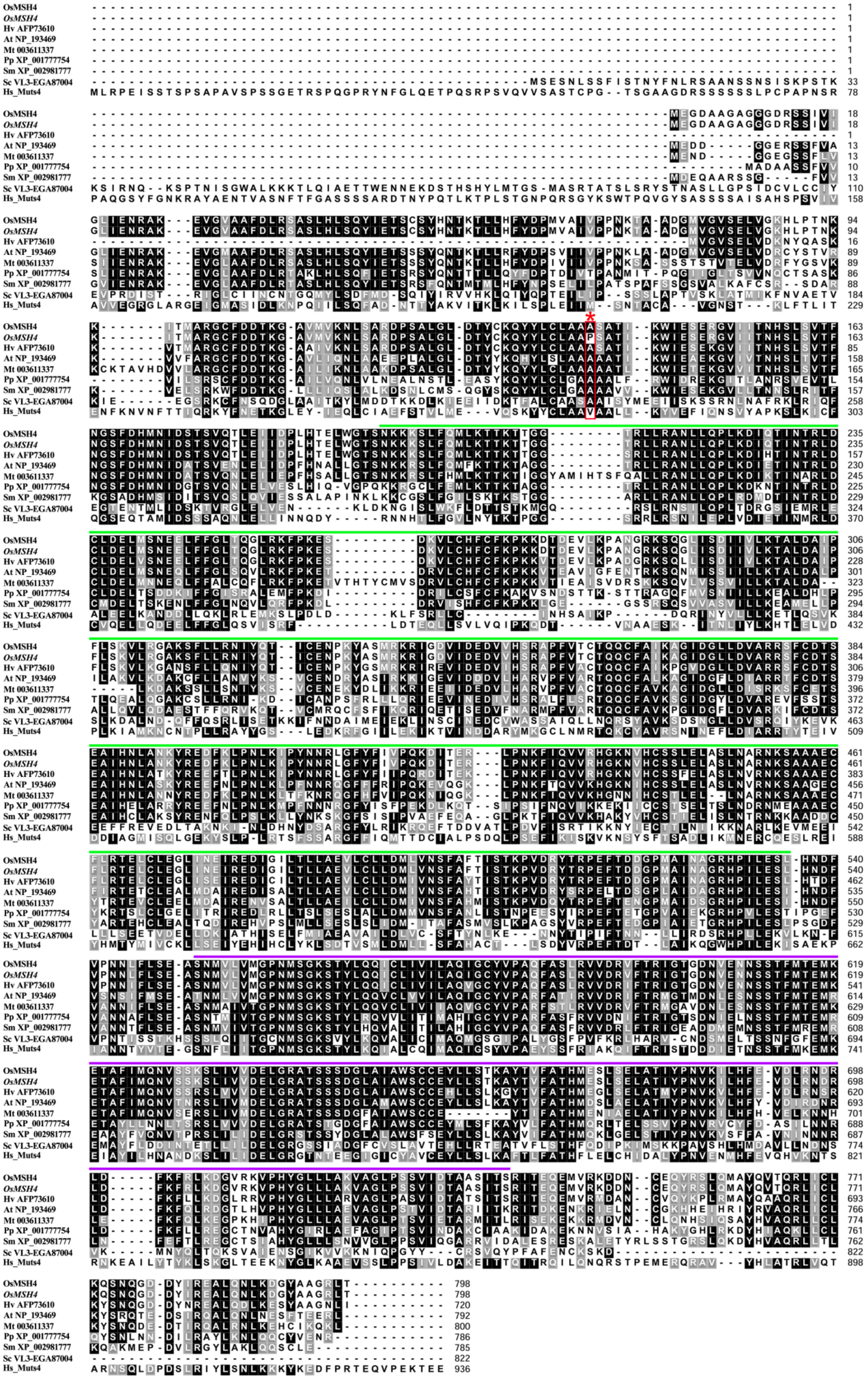
OsMSH4
OsMSH4
Hv AFP73610
At NP_193469
Mt 003611337
Pp XP_001777754
Sm XP_002981777
Sc VL3-EGA87004
Hs_Muts4

Supplementary Figure S6 Multiple sequence alignment of the amino acid sequence of OsMSH4 and its homologs. Red asterisk indicates the mutated amino acid, and the predicted MUTSd and MUTSac domains are shown as green and purple lines respectively. The sequences were from the following organisms: Hv, *Hordeum vulgare*; At, *Arabidopsis thaliana*; Mt, *Medicago truncatula*; Pp, *Physcomitrella patens*; Sm, *Selaginella moellendorffii*; Sc, *Saccharomyces cerevisiae*; Hs, *Homo sapiens*.

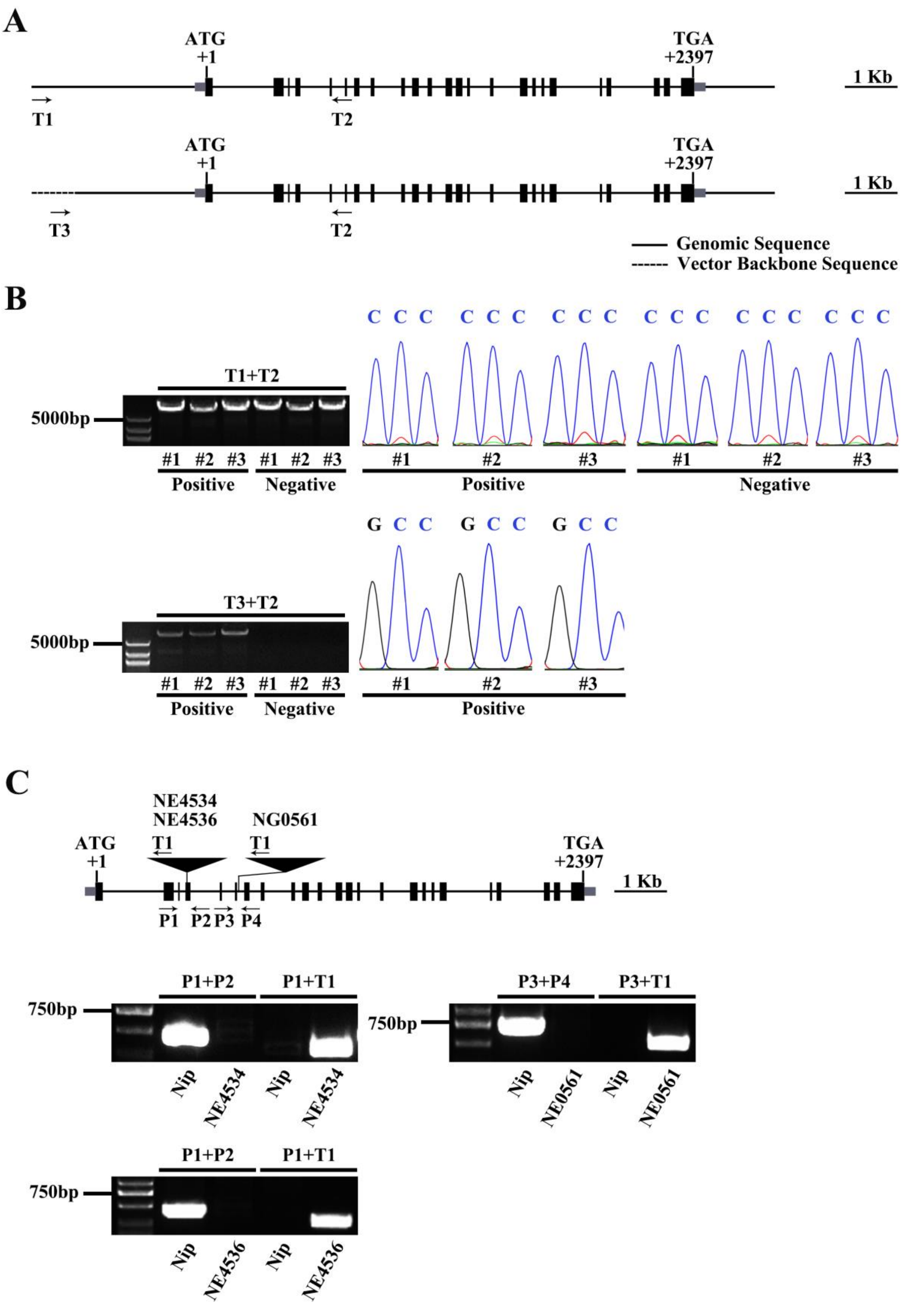

Supplementary Figure S7 Analysis of transgenic complementation and three lines of *TOS17* insertions.

(A) Schematic structure of *OsMSH4* and the positions of detection primers. T1 and T2 located on the genomic sequence while T3 on the vector backbone sequence. (B) Genotyping and sequence analysis of positive and negative transgenic T_1 plants. (C)

The position of *TOS17* insertions in three lines.

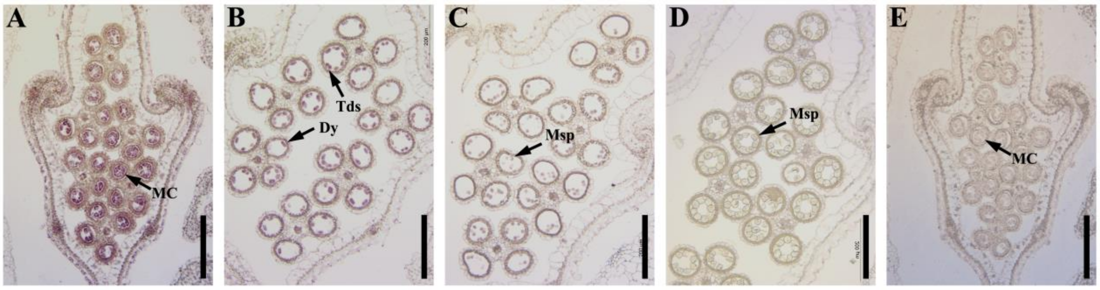

Supplementary Figure S8 *In suit* hybridization assays of *OsMSH4* at different stages of anther development.

(A) Transverse sections of an anther at the microspore mother cell stage. (B) Dyad stage. (C) Early microspore stage. (D) Late microspore stage. (E) Negative controls with the sense probe at the microspore mother cell stage. Bars = 200 μm.

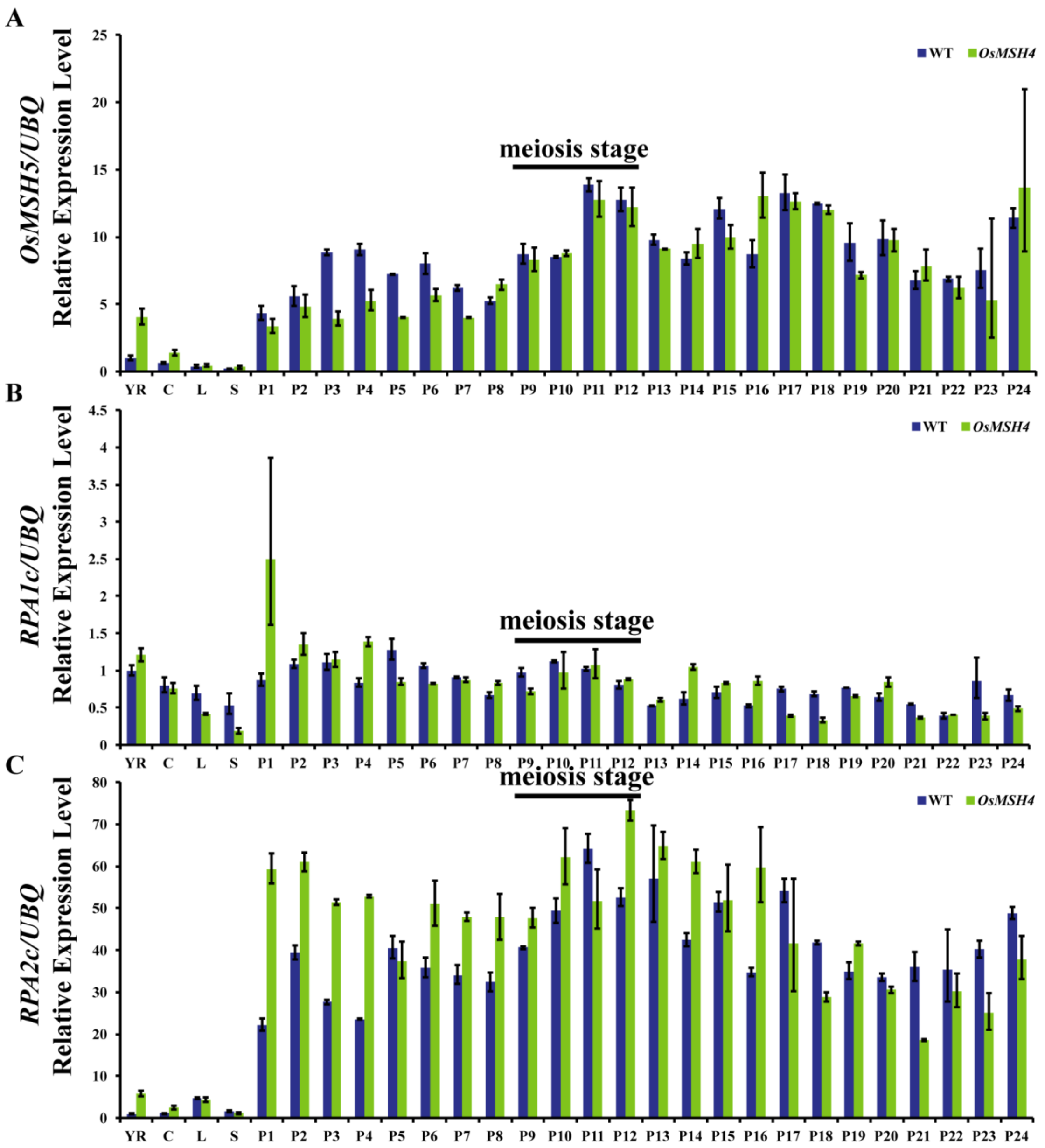

Supplementary Figure S9 Temporal and spatial expression pattern analyses of *OsMSH5*, *OsRPA1c* and *OsRPA2c* by quantitative RT-PCR. YR, young roots; C, mature culms; L, mature leaves; S, mature sheaths; P1-P24, spikelet length, from 1 to 24 cm.

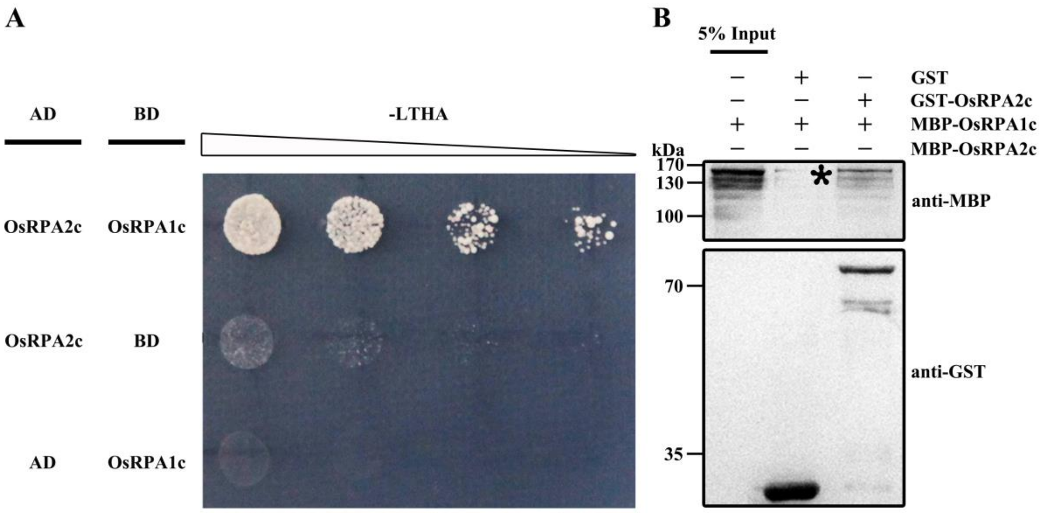

Supplementary Figure S10 The interaction between OsRPA1c and OsRPA2c.

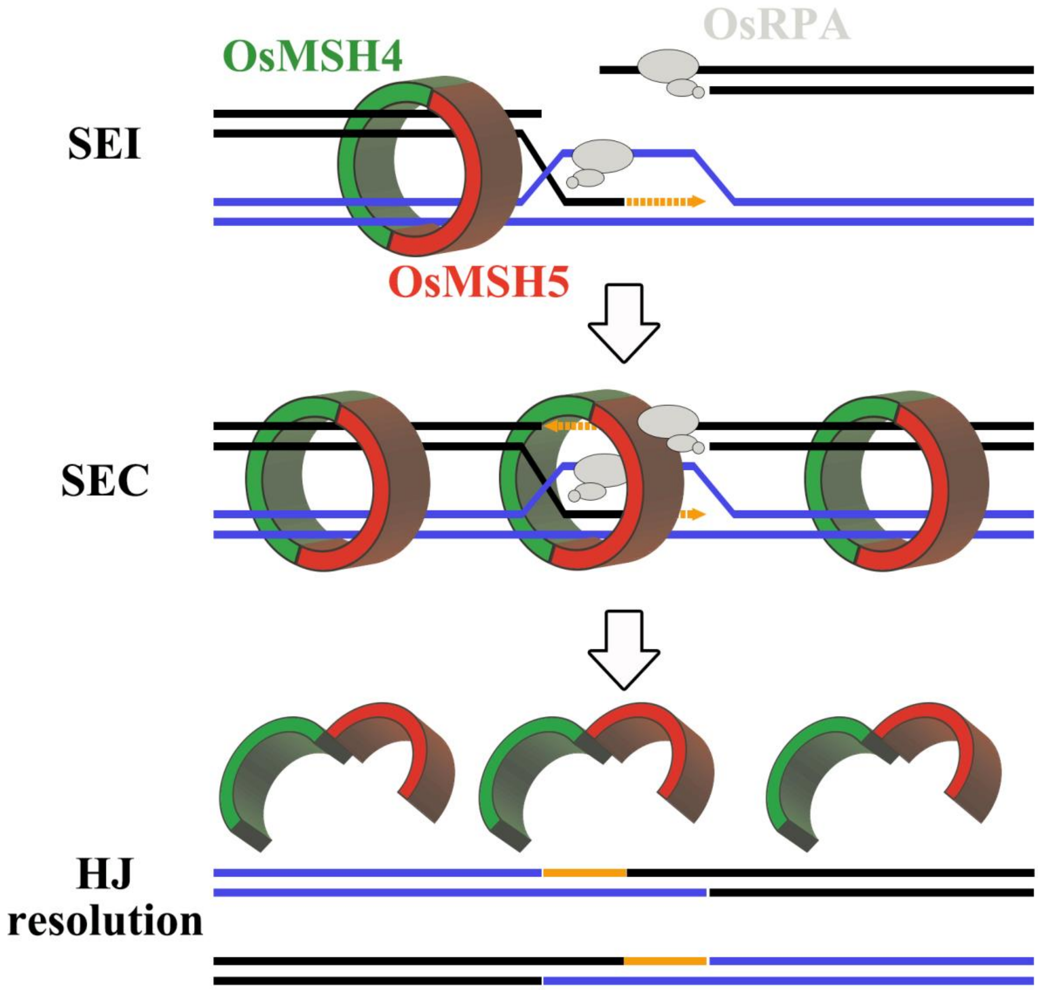

Supplementary Figure S11 A schematic model depicting the OsMSH4/OsMSH5 heterodimer interacting with the OsRPA heterotrimeric complex during second-end capture to regulate crossover formation during meiosis I.

Supplementary Table S1. Morphological traits comparison of the *Osmsh4* mutant with the 6537 and 6537W.

Genotype	Plant height (cm)	Tiller number	Panicle length (cm)	Primary branch number	Second branch number	Spikelet fertility (%)	Spikelet number	Flag leaf Width (cm)	Flag leaf length (cm)
6537	94.0±2.2	8.3±1.2	23.3±1.3	13.4±1.4	24.8±3.6	24.1±5.8	143.6±16.7	1.1±0.1	40.4±1.4
6537W	109.8±4.5	10.4±2.3	25.0±1.6	11.4±1.6	32.3±3.8	82.0±2.1	217.0±22.1	1.4±0.1	31.6±3.6
Osmsh4	94.6±2.4	11.0±1.2	23.9±0.7	11.2±0.9	31.4±4.9	0	182.2±25.2	1.5±0.1	30.0±2.9

Supplementary Table S2. Gene annotation of eight putative genes within the 63 kb regions based on BAC clones of cv. Nipponbare.

No.	Predicted genes	Gene annotation
1	LOC_Os07g30180	Transposon protein
2	LOC_Os07g30190	Retrotransposon protein
3	LOC_Os07g30200	tRNA synthetases class II domain containing protein
4	LOC_Os07g30210	Integral membrane protein DUF6 domain containing protein
5	LOC_Os07g30220	Expressed protein
6	LOC_Os07g30234	Expressed protein
7	LOC_Os07g30240	mutS family domain IV containing protein
8	LOC_Os07g30250	RFT1

Supplementary Table S3. Primers used in this study.

Prime name	Prime sequence (5'-3')	Description
M3-F	TTCTGTCTCACGCTGGATTG	Mapping
M3-R	AGCTGCGTACGTGATGAATG	
M5-F	ACATCCAAACCGGATTAACG	
M5-R	GAATAAGACGAGTGGTCAAACG	
M6-F	CAGCCATCTATCTCATCACCAA	
M6-R	GATGCGTCTGGGTCATCC	
M10-F	TGAAATTGTGTAATTCTGCCACA	
M10-R	CCCATGTGAGTTGCTCTCTG	
M11-F	CAACAACGAGACCGCACTTA	
M11-R	CGACGTCATGGGAGTCTTC	
M12-F	TTATTGCTCACCCTTGCACA	
M12-R	GCATATGCTAAGAAACAAAAGCAA	
M13-F	GCGTCACTGACAGTCGTTGT	
M13-R	ACTGTAGGCGGTTGACCACT	
1305-*OsMSH4*-Genomic-F	ATCCTCTAGAGTCGACATTTGGATCGGATTTGATCTCGTT	*OsMSH4* complementation
1305-*OsMSH4*-Genomic-R	ATGCCTGCAGGTCGACGGTTCGGTTGATGTTTCACATAGTTT	
O4-Insitu-F	TGGCTGCTTTGATGACACAAAG	*OsMSH4 in situ*
O4-Insitu-R	GACGAGTATTGATTGTTTGAATGTCTT	
O4-GUS-F	CCATGATTACGAATTCAAGAACACTGAACCATACAAATACAAGCAA	$OsMSH4_{pro}$: *GUS*
O4-GUS-R	CTCAGATCTACCATGGGCGACGGTGGAATCCGAAGC	
TR2-O4-GFP-XBAI-F	CACCAAATCGTCTAGAATGGAGGGCGACGCCG	OsMSH4 subcellular localization
TR2-O4-GFP-XBAI-R	TCGAGACGTCTCTAGATGTCAAGCGGCCTGCAGC	
SZ-*OsMSH5*-CDS-AD-F	GGAGGCCAGTGAATTCATGGACGAGGAAGAGGAGGAG	Yeast two-hybrid assay

SZ-*OsMSH5*-CDS-AD-R	CGAGCTCGATGGATCCGGACGGAAAAACTTCCTGGAA	
SZ-*OsMSH4*-CDS-BK-F	CATGGAGGCCGAATTCATGGAGGGCGACGCCGCCG	
SZ-*OMSH4*-CDS-BK-R	GCAGGTCGACGGATCCTGTCAAGCGGCCTGCAGCATAGCC	
SZ-*OsMSH4*-CDS(1-194)-BK-F	CATGGAGGCCGAATTCATGGAGGGCGACGCCGCCG	
SZ-*OsMSH4*-CDS(1-194)-BK-R	GCAGGTCGACGGATCCGCTAGTTCCCCACAGTTCAGTATGA	
SZ-*OsMSH4*-CDS(1-300)-BK-F	CATGGAGGCCGAATTCATGGAGGGCGACGCCGCCG	
SZ-*OMSH4*-CDS(1-300)-BK-R	GCAGGTCGACGGATCCTGTTTTTAGAACAATAATGTCAGAA	
SZ-*OsMSH4*-CDS(1-536)-BK-F	CATGGAGGCCGAATTCATGGAGGGCGACGCCGCCG	
SZ-*OsMSH4*-CDS(1-536)-BK-R	GCAGGTCGACGGATCCCAAGCTCTCAAGAATAGGATGCCTT	
SZ-*OsMSH4*-CDS(739-798)-BK-F	CATGGAGGCCGAATTCCGGATCACAGAACAGGAAATGGTGA	
SZ-*OsMSH4*-CDS(739-798)-BK-R	GCAGGTCGACGGATCCTCATGTCAAGCGGCCTGCAGCATAG	
SZ-*OsMSH4*-CDS(551-798)-BK-F	CATGGAGGCCGAATTCTCTAATATGGTTCTTGTCATGGGGC	
SZ-*OsMSH4*-CDS(551-798)-BK-R	GCAGGTCGACGGATCCTCATGTCAAGCGGCCTGCAGCATAG	
SZ-*OsMSH4*-CDS(195-798)-BK-F	CATGGAGGCCGAATTCAACAAAAAGAAAAGTCTCTTTCAGAT	
SZ-*OsMSH4*-CDS(195-798)-BK-R	GCAGGTCGACGGATCCTCATGTCAAGCGGCCTGCAGCATAG	
BD-*OsRPA1c*-F	CATGGAGGCCGAATTCATGGAGCCGCAGCTGACG	
BD-*OsRPA1c*-R	GCAGGTCGACGGATCCTCAGTAGCCACCAACAAAGGATTG	
BD-*OsRPA2c*-F	CATGGAGGCCGAATTCATGGCCGCGGCCGCGT	
BD-*OsRPA2c*-R	GCAGGTCGACGGATCCTCAACCATTACACATAGACTTGACATGAT	
BD-*OsRPA1a*-F	CATGGAGGCCGAATTCATGGCGATGGCGAGGCTGAC	
BD-*OsRPA1a*-R	GGATCCCCGGGAATTCCTAATGTAGCGCCGAGGACT	
BD-*OsRPA2b*-F	CATGGAGGCCGAATTCATGTACGGCGTCGGCGTCGG	
BD-*OsRPA2b*-R	GGATCCCCGGGAATTCTCAACCATTCATTGTGGACT	
O4-PMAL-C2X-F	AAGGATTTCAGAATTCATGGAGGGCGACGCCGCCG	Protein expression in *E.Coli*
O4-PMAL-C2X-R	TAGAGGATCCGAATTTCATGTCAAGCGGCCTGCAGCATAG	
O5-PGEX-4T-1-F	CCCGGGTCGACTCGAGATGGACGAGGAAGAGGAGGAG	

O5-PGEX-4T-1-R	GATGCGGCCGCTCGAGCTAGGACGGAAAAACTTCCTGGAAG	
OsRPA1C-PMAL-C2X-F	AAGGATTTCAGAATTCATGGAGCCGCAGCTGACG	
OsRPA1C-PMAL-C2X-R	CGACTCTAGAGGATCCTCAGTAGCCACCAACAAAGGATTG	
OsRPA2C-PMAL-C2X-F	AAGGATTTCAGAATTCATGGCCGCGGCCGCGT	
OsRPA2C-PMAL-C2X-R	CGACTCTAGAGGATCCTCAACCATTACACATAGACTTGACATGAT	
OsRPA2C-PGEX-4T-1-F	CCCGGGTCGACTCGAGATGGCCGCGGCCGCGT	
OsRPA2C-PGEX-4T-1-R	GATGCGGCCGCTCGAGTCAACCATTACACATAGACTTGACATGAT	
OsRPA1a-PMAL-C2X-F	AAGGATTTCAGAATTCATGGCGATGGCGAGGCTGAC	
OsRPA1a-PMAL-C2X-R	TAGAGGATCCGAATTCTAATGTAGCGCCGAGGACT	
OsRPA2b-PMAL-C2X-F	AAGGATTTCAGAATTCATGTACGGCGTCGGCGTCGG	
OsRPA2b-PMAL-C2X-R	TTGCCTGCAGGTCGACTCAACCATTCATTGTGGACT	
DL-O4-F	TGCCAGGAATAAGTCTGCTG	qRT-PCR
DL-O4-R	TAATACTTCGGCAAGCAACG	
DL-O5-F	CCAACAATACCAGGATGCAG	
DL-O5-R	TGATCTGCCAATTCCATGTT	
DL-*OsRPA1c*-F	CCTACTTGGAGCGATTGACA	
DL-*OsRPA1c*-R	TTCACGCCACCAGTATTCAT	
DL-*OsRPA2c*-F	GGCAATTTCCTCCATCAGTT	
DL-*OsRPA2c*-R	CAGAAGAAGCCGCATGATTA	
